# Bilateral fracture of the olecranon in a young footballer

**DOI:** 10.11604/pamj.2014.17.52.3703

**Published:** 2014-01-24

**Authors:** Monsef Boufettal, Rachid El Zanati

**Affiliations:** 1 Orthopedic Surgery Department of Ibn Sina hospital, Rabat, Morocco

**Keywords:** Fracture, olecranon, footballer

## Image in medicine

A 24-year-oldathlete presented following a fall onto an outstretched hand during a football match, total functional impotence of both upper limbs with swollen elbows, pain, without skin opening or motor deficit (A). Radiography of both elbows was objectified a fracture of the both olecranon (B). The patient was treated by tension-band wiring fixation with precocious postoperative rehabilitation (C). Two months after surgery, we note a consolidation of the fracture with satisfactory mobility of both elbows. Olecranon fractures may be caused by direct injury to the posterior part of the elbow joint or indirectly by forces generated within the triceps muscle during a fall on a partially flexed elbow. The clinical picture is obvious and conventional radiographs are usually sufficient to depict the lesion and the potential associated injuries. The bilateral form is very rarely described in the literature. The goals of treating olecranon fractures are anatomic restoration of the articular surface, repair of the elbow extensor mechanism, restoration of joint stability and motion, and prevention of stiffness and other complications. Treatment options include immobilization, surgical reduction and fixation with tension-band wiring or plate osteosynthesis. The active mobilization after surgery will restore the patient to normal functions as early as possible.

**Figure 1:**
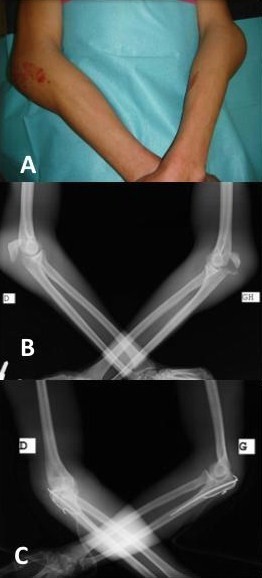
A) deformation of two elbows; B) Radiography objectified a fracture of the both olecranon; C) surgical treatment by Tension-band wiring fixation

